# Severe visual loss by inhalation of methanol

**DOI:** 10.22336/rjo.2021.34

**Published:** 2021

**Authors:** Belén Figuerola, Angi Mendoza, Mercedes Roca, Javier Lacorzana

**Affiliations:** * Department of Ophthalmology, General University Hospital, Alicante, Spain; ** Department of Ophthalmology, Virgen de las Nieves University Hospital, Granada, Spain; ** Doctoral Program in Clinical Medicine and Public Health, University of Granada, Granada, Spain

**Keywords:** inhalational methanol, methanol intoxication, Toxic Optic neuropathy, Optical Coherence Tomography, therapeutic management

## Abstract

**Purpose:** To describe a clinical case of toxic optic neuropathy with severe visual loss caused by inhalation abuse of methanol products.

**Method:** A 25-year-old male student was admitted to the emergency department with an acute bilateral visual loss and headaches, nausea, and cold sweats. A complete clinical and ophthalmologic examination was performed.

**Results:** On ophthalmic examination, visual acuity (VA) was light perception in the right eye (RE) and no light perception in the left eye (LE). Pupillary examinations demonstrated dilated, non-reactive pupils. An arterial blood gas analysis showed systemic metabolic acidosis with a pH of 7.23 and Gap anion elevated. Consequently, these results were enough to provide a substantial suspicion of methanol toxicity and start the treatment. 72 hours after, he confessed that he had been inhaling methanol-based solvent for eight years.

**Conclusions:** Methanol-induced toxicity can cause a non-reversible toxic optic neuropathy. Blood acidemia with Gap anion elevated and a suspicious fundus ophthalmic examination allows a fast diagnosis. A quick treatment based on dialysis, intravenous ethanol, sodium bicarbonate, vitamin B12, and intravenous methylprednisolone slows the secondary intoxication damages. We presented herein a procedure to identify and manage toxic optic neuropathy caused by methanol inhalation.

**Abbreviations:** VA = Visual Acuity, RE = right eye, LE = left eye, OCT = Optical Coherence Tomography, RNFL = Retinal Nerve Fiber Layer, CT = computed tomography, MRI = magnetic resonance imaging, VEPs = visual evoked potentials

## Introduction

Methanol is a non-drinking type of alcohol. It is a widely present chemical in many household items (paint remover, dyes, cologne, solvents, or antifreeze). It is a colorless, volatile, and flammable liquid. Unlike ethanol, methanol is poisonous for human consumption. It has been used for illegal activities for a long time, such as the occasional adulteration (primarily fraudulent) of alcoholic beverages like wine [**1**].

Methanol intoxication is very dangerous, and there are reported cases of metabolic acidosis, severe visual dysfunction, permanent neurological dysfunction, and death [**2**]. The most frequent form of intoxication is oral. However, other non-oral forms of intoxication can cause the same effects. We presented herein a case of a patient with a methanol addiction history who presented a severe loss of acute vision after an intentional chronic inhalation of a methanol-based paint remover.

## Case report

A 25-year-old male student was admitted to the emergency department with an initial complaint of blurred vision with 12 hours of evolution. The night before the symptom onset, he drank alcoholic beverages (beers) with his roommate from a local supermarket. He developed nausea, vomiting, and an intense headache. The following day, he woke up with generalized malaise, photophobia, and blurred vision. He denied taking any other drugs. Incoherently, his roommate, who consumed the same drinks, was asymptomatic.

The patient was conscious, cooperative, and well oriented to time and place on the general examination. Neurological, respiratory, and abdominal examinations were routinely performed. Also, electrocardiography, chest radiography, and urinalysis toxics presented standard values. However, an arterial blood gas analysis showed systemic metabolic acidosis with a pH of 7.23 and an elevated anion gap. 

**Fig. 1:**
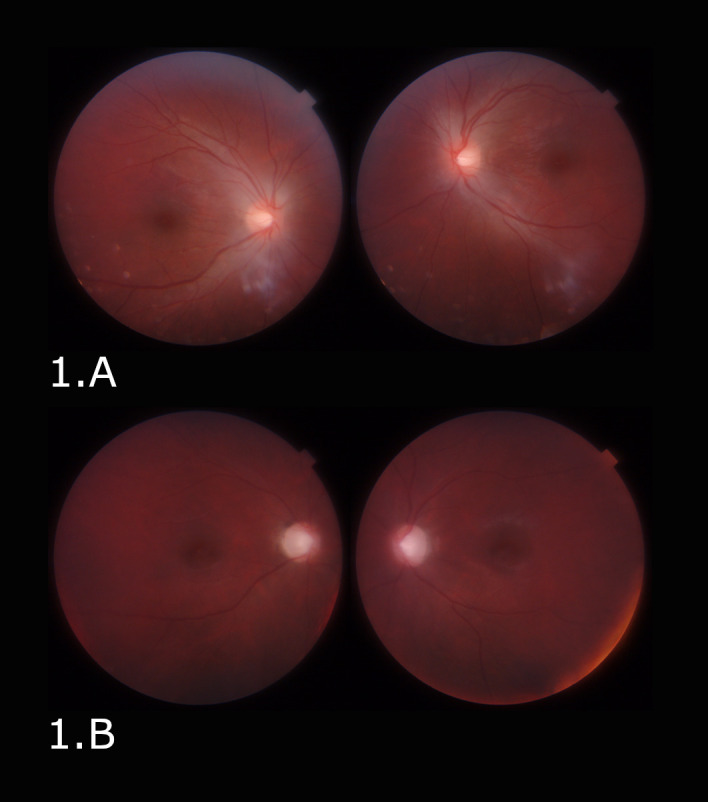
Fundus examination. **A.** Optic discs were hyperemic and edematous. **B.** Optic discs are phallic with atrophic signs

On ophthalmic examination, visual acuity (VA) was light perception in the right eye (RE) and no light perception in the left eye (LE). Pupillary examinations demonstrated dilated, non-reactive pupils. In the fundus examination, the optic discs were hyperemic, poorly delimited, and a peripapillary retinal edema was present. In the Optical Coherence Tomography (OCT), the retinal nerve fiber layer (RNFL) showed thickening (**Fig. 1 A**, **3A, 3B**). 

Based on the symptoms presented and history of alcohol consumption, the patient was admitted with bilateral toxic optic neuritis due to a suspected methanol poisoning. He was treated with intravenous ethanol, sodium bicarbonate, and vitamin B12. Moreover, he required dialysis to treat his metabolic acidosis. To treat his visual loss due to optic neuritis, intravenous methylprednisolone (1000 mg for three days, 500 mg for three days) and vitamin B complex and folic acid were administered.

**Fig. 2:**
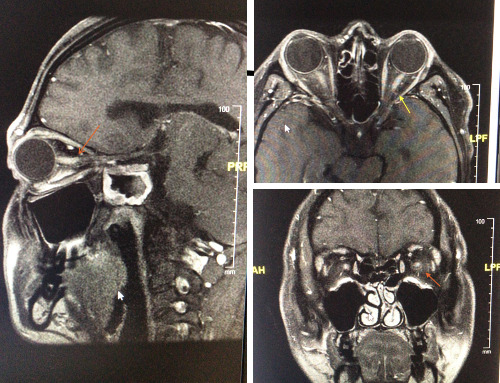
MRI: mild bilateral contrast enhancement on the contrast-enhanced images in the optic nerves’ retrobulbar segment (arrows)

A computed tomography (CT) scan and a magnetic resonance imaging (MRI) were performed to assess the nervous system and the internal anatomical structures. CT-scan was normal and his optical nerves presented a normal morphology and thickness. Nevertheless, a mild bilateral contrast enhancement on the contrast-enhanced images in the optic nerves’ retrobulbar segment was detected on the MRI (**Fig. 2**). 

His serum methanol level was abnormal (15.5 mg/ dl). To explain this, the patient confessed that he had been inhaling a methanol-based solvent for eight years, and his last ingestion was four days before the blood test. Furthermore, in the last few months, he had increased the frequency of inhalation. This solvent was available at his local supermarket.

**Fig. 3:**
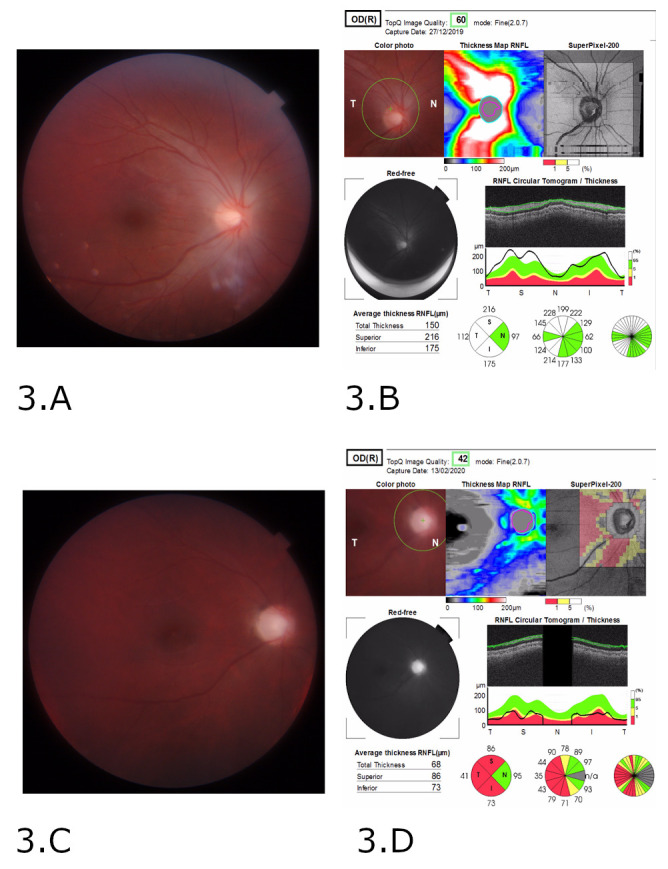
Fundus examination in the right eye. **A.** Optic discs were hyperemic and edematous. **B.** Optical Coherence Tomography, RNFL showed thickening in the right eye. **C.** Optic disc in the right eye is phallic with atrophic signs. **D.** Optical Coherence Tomography, RNFL is thinning in the right eye

Visual evoked potentials (VEPs) with a checkerboard pattern and white light flash were recorded seven days after the onset of visual symptoms, showing an axonal type alteration with significant damage in macular fascicules in both eyes. Seven weeks following the methanol intoxication, the VA improved (0.1 RE and 0.05 LE), although he had a persistent big central “blind spot” in both eyes. In the fundus examination, the optic discs were phallic with atrophic peripapillary retinal signs. In the OCT, the RNFL showed thinning, preserving only the nasal sector fibers (**Fig. 1 B**, **3C, D**).

## Discussion

Methanol is metabolized in the human body by enzymes alcohol dehydrogenase into formaldehyde. The resulting outcome then gets metabolized by aldehyde dehydrogenase into formic acid. Formic acid is toxic and it inhibits the mitochondrial cytochrome oxidase causing tissue hypoxia, and lactic acid [**3**]. 

Ocular toxicity is thought to be due to the formate anion’s direct effect, which is believed to cause optic neuritis, and thereupon loss of vision. This happens mainly because of the interruption of the mitochondrial function in the retrolaminar or retrobulbar portion of the optic nerve. The initial ophthalmologic symptoms of methanol toxicity appear 6-48 h post-ingestion. However, the onset of symptoms after methanol inhalation is not defined. It depends on the exposure duration, as well as the concentration level in the space. The severity of symptoms varies widely from mild progressive and painless decreases in vision to no-light perception vision, dyschromatopsia, scotomata, and photophobia [**4**].

In our case, on his third day of hospitalization, the patient admitted that he sporadically, although lately more frequently, inhaled methanol from methanol-based solvent products that he acquired from a local supermarket. This hospitalization had been his only intoxication episode after eight years of addiction. A specific methanol intoxication diagnosis could have been obtained with the measurement of this alcohol in the blood. However, the results from such tests in our clinical practice were dependent on an external laboratory. In the absence of a clinical history, our diagnosis was supported by acidemia with elevated anion gap [**5**]. Our initial diagnosis was difficult because the patient hesitated to acknowledge his chronic addiction. The laboratory findings were important for the intoxication treatment and the confirmed etiological diagnosis. 

Cases of methanol poisoning caused by oral ingestion can experience an acute attack following just consumption or the misuse of methanol, with obvious systemic symptoms. Most of the patients typically seek medical attention, and they might be cured or partially improved after being administered an appropriate treatment. 

Many reports have been published on poisoning due to methanol ingestion. However, the inhalation route, which is less reported, is also an important way of exposure [**6**]. There have been reports of inhalational methanol poisoning suggesting a benign course; other cases leading to severe toxicity and blindness were similar to the findings in our case [**7**].

The inhalation of methanol to the poisonous dose might be a chronic accumulation of methanol and its metabolites in the body before acute onset. When there are prominent visual symptoms, they may urge patients to visit an ophthalmologist or neuro-ophthalmologist repeatedly. Inhalational abusers of methanol-containing products should have serum methanol levels and electrolytes measured, but an average anion gap does not exclude potentially significant serum methanol or formate levels. Moreover, all patients should receive a fundoscopic exam, have the VA measured, and be monitored for an anion gap acidosis. 

The routine treatment regimens include the use of ethanol, folinic acid, sodium bicarbonate, and hemodialysis [**8**]. These treatments mainly prevent formic acid and further toxicity but do not affect established ocular inflammation. Some studies reported that intravenous methylprednisolone improved in different degrees in a year follow-up, but not satisfactorily [**9**]. Our patient was treated with intravenous methylprednisolone, followed by oral prednisolone and B vitamins. Nevertheless, compared to the visual functions before treatment, the improved visual function was not satisfactory. Since the patient’s work capacity was drastically damaged by the detrimental effects on the visual system and the peripheral nervous system, the ophthalmologic treatment consisted of multidisciplinary and psychological support. Weekly follow-ups provided fundamental clinical support ambulatory. Progressive visual loss was substantial, and the campimetry restriction was significantly reduced in both eyes due to peripheral scotoma.

## Conclusion

In conclusion, the aim of this case report was to highlight the importance of fast handling when suspecting methanol intoxication, being the multidisciplinary follow-up essential to get good results. In our case, the outcome of the chronic intoxication was unfavorable despite the systemic treatment. 

**Conflict of Interest statement**

Authors state no conflict of interest.

**Informed Consent and Human and Animal Rights statement**

Informed consent has been obtained from all individuals included in this study.

**Authorization for the use of human subjects**

Ethical approval: The research related to human use complies with all the relevant national regulations, institutional policies, is in accordance with the tenets of the Helsinki Declaration, and has been approved by the review board of General University Hospital, Alicante, Spain.

**Acknowledgements**

None.

**Sources of Funding**

None.

**Disclosures**

None.
